# Expression and localisation of Akt-1, Akt-2 and Akt-3 correlate with clinical outcome of prostate cancer patients

**DOI:** 10.1038/sj.bjc.6603184

**Published:** 2006-05-23

**Authors:** C Le Page, I H Koumakpayi, M Alam-Fahmy, A-M Mes-Masson, F Saad

**Affiliations:** 1 Département d’urologie, Centre de recherche du Centre Hospitalier de l’Université de Montréal (CR-CHUM) and Institut du cancer de Montréal, Hôpital Notre-Dame, 1560 rue Sherbrooke Est, Montreal, Quebec, Canada H2L4M1; 2 Département de médecine, Université de Montréal, Montreal, Quebec, Canada H3C3J7; 3 Département d’urologie, Université de Montréal, Montreal, Quebec, Canada H3C3J7

**Keywords:** prostate cancer, Akt, PSA recurrence, prognosis, immunohistochemistry

## Abstract

We investigated the correlation between the expression and localisation of Akt-1, Akt-2, Akt-3, phospho-Akt proteins and the clinicopathological parameters in 63 prostate cancer specimens. More than 60% of cancerous tissues overexpressed Akt-1, Akt-2 or Akt-3. Cytoplasmic Akt-1 expression was correlated with a higher risk of postoperative prostate-specific antigen (PSA) recurrence and shorter PSA recurrence interval. Cytoplasmic Akt-2 did not show any significant correlation with clinicopathological parameters predicting outcomes. Cytoplasmic Akt-3 was associated with hormone-refractory disease progression and extracapsular invasion. Nuclear Akt-1 and Akt-2 expression were correlated with favourable outcome parameters such as absence of lymph node and perineural invasion. Kaplan–Meier analysis and Cox regression model also showed that Akt-1 and Akt-2, but not Akt-3 or phospho-Akt was associated with a significantly higher risk of PSA recurrence. In contrast, nuclear Akt-1 was significantly associated with a lower risk of PSA recurrence. Multivariate analysis revealed that clinical stage, Gleason score and the combined cytoplasmic nuclear Akt-1 marker in cancerous tissues were significant independent prognostic factors of PSA recurrence. This is the first report demonstrating in patients with prostate cancer and the particular role of Akt-1 isoform expression as a prognostic marker depending of its localisation.

In mammals, three genes have been identified encoding for the isoforms of the serine/threonine protein kinase B family, or Akt. Members of this family, Akt-1, Akt-2 and Akt-3, share 80% homology. Akt is a signalling component of the tyrosine kinase growth factor receptors and G-coupled receptors. These receptors engage the heterodimeric phosphoinositol 3-kinase (PI3K), which phosphorylates phosphatidylinositol-4, 5-bisphosphate to convert PIP2 to PIP3. PIP3 levels are tightly regulated by the phosphatase PTEN, which removes phosphate from the 3-OH position. Then PIP3 recruits other signalling components such as Akt and PDK. PDK then phosphorylates and activates Akt, which in turn regulates downstream signalling proteins involved in cell survival, cell growth, cell cycle progression and apoptotic response (Vanhaesebroeck and Alessi, 2000). Akt/PI3K signalling is often disregulated in human cancer mainly due to constitutive activation of growth factor receptors and loss of PTEN function. Accumulating evidence shows a central role of Akt in tumour development and response to cancer treatment (Wendel *et al*, 2004).

Although there are a limited number of studies, it seems that all three isoforms are ubiquitously expressed but the level of expression of each isoform may differ among tissues thus modulating in part their biological activities. For example, Akt-1 is more highly expressed in tissues such as brain, thymus and lungs, whereas Akt-2 gene amplification has been found in ovarian, breast and pancreas tumours where it has been implicated in the process of invasion and metastasis (Bellacosa *et al*, 1995, 2005). Akt-3 is predominantly expressed in brain, heart and kidney and seems to be responsible for the survival of melanoma cells (Stahl *et al*, 2004; Bellacosa *et al*, 2005).

In prostate cancer cell lines, the constitutive activity of Akt is mainly due to the constitutive activation of EGFR (Le Page *et al*, 2005) and PDGFR (Dolloff *et al*, 2005) in association with a loss of PTEN (Dong *et al*, 1998; Gray *et al*, 1998; Suzuki *et al*, 1998). This activity has been associated with advance cancer stage, resistance to apoptosis through NF-*κ*B activation (Li and Sarkar, 2002; Raj *et al*, 2004), downregulation of PTEN and P27 (Graff *et al*, 2000; Murillo *et al*, 2001; Ayala *et al*, 2004). In clinical specimens, the overexpression and activation of Akt has been associated with high preoperative prostate-specific antigen (PSA) level, higher Gleason grades and shorter disease relapse (Malik *et al*, 2002; Liao *et al*, 2003; Ayala *et al*, 2004; Kreisberg *et al*, 2004). However, none of these studies have investigated which isoform(s) is associated with these phenomena. In fact, the specific expression and role of Akt-1, -2 and -3 in prostate cancer tissues is still scant. Only one study has investigated the mRNA level of the isoforms (Zinda *et al*, 2001) but did not examine the protein expression or the activity of each isoform. From studies examining prostate cancer cell lines, some evidence suggests that Akt isoforms are differentially regulated in prostate cancer cells and may have different roles. For example, Akt-3 is not expressed in androgen receptor positive prostate cell lines and no constitutive activation of Akt-2 has been observed in prostate cancer cell lines while Akt-1 is constitutively activated in most prostate cancer cell lines (Nakatani *et al*, 1999). As it is still unknown if the same pattern occurs in prostate cancer tissues, analysis of Akt expression is necessary. A better knowledge of Akt expression and its role will clarify their redundant or complementary function in cancer progression.

Here, we examined the expression and localisation of the three isoforms of Akt on tissue arrays containing 373 tissue cores from 63 prostate cancer patients. We also analysed the correlation and association between expression of each isoform, the presence of Akt activity and clinical parameters in order to determine whether the three Akt isoforms are associated with specific parameters in prostate cancer progression.

## MATERIALS AND METHODS

### Patient cohort

Paraffin-embeded human primary prostate cancer specimens from patients having provided informed consent and operated from 1993 to 2000 were reviewed. In total, 64 specimens were included in our study to create a tissue array. Criteria for the retrospective cohort study to create the tissue arrays were: no preoperative treatment was used in this cohort, and all cases had clinical follow-up of at least 5 years or until death. Patients were followed for an average range of 72 months. No age difference was observed between the group of patients who relapsed and the group that did not. Preoperative PSA level was available for 62 patients. Postoperative PSA was available for all patients. PSA nonfailure was defined as PSA remaining below 0.3 ng ml^−1^ after radical prostatectomy. Recurrence-free interval was defined as the time between date of surgery and the date of first PSA increase above 0.3 ng ml^−1^. The final staging, grading and histologic diagnosis was based on the Hospital Notre-Dame (Montreal, QC, Canada) pathology report in agreement with the review from an independent pathologist. Ethics approval was obtained from the local IRB committee.

### Tissue arrays, immunohistochemistry and scoring

Tissue arrays containing a total of 384 cores of prostate tissues (Table 1) were built and used for IHC studies. One normal and two cancerous cores per patient were spotted on one array and a duplicate of this array was made, making a total of six cores per patients. An expert pathologist determine the cancerous, non-neoplasic prostatic epithelium and PIN areas reviewed H&E-stained arrays. Eleven cores that contained neither cancerous tissues nor non-neoplasic adjacent tissues were not considered for further analysis. The final analysis contained 373 tissue cores representing 63 patients. No patient was represented by less than two cores.

The following amounts and antibodies were used in immunohistochemistry (IHC): a 
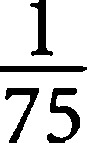
 dilution of anti-Akt-1 (D17), a 
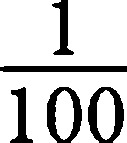
 dilution of anti-Akt-2 (F7), a 
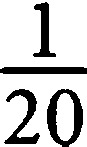
 dilution of Akt-3 (M14) (all from Santa Cruz Biotechnology, CA, USA) and a 
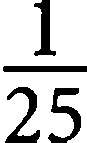
 dilution of phospho-Akt (#9277) (Cell Signaling, Danvers, MA, USA). Briefly, tissue arrays were deparaffinised in toluene and rehydrated in a gradient of ethanol. Subsequently, endogenous peroxidase activity was quenched by treatment with 0.3% H_2_O_2_/methanol. To unmask antigen the slides were submerged in 95°C citrate solution (pH 6.0) for 15 min. The tissues were blocked for 15 min with a protein-blocking serum-free reagent (Dako Cytomation Inc., Mississauga, ON, Canada) and incubated with the different antibodies for 60 min at RT in a humid chamber. The optimal concentration for each primary antibody was determined by serial dilutions. The arrays were then incubated with a secondary biotinylated antibody (DakoCytomation Inc., Mississauga, ON, Canada) for 15 min followed by incubation with a streptavidin–peroxidase complex (Dako Diagnostics Canada Inc.). Reaction products were developed using diaminobenzidine (brown stain) containing 0.3% H_2_O_2_ as a substrate for peroxidase. Nuclei were counterstained with diluted haematoxylin (blue stain). Tumour sections were inspected at × 20 and × 40 magnification. Epithelial zones were scored according to the intensity of staining of the cytoplasm, nucleus or membrane (value of 0 for absence, 1 for weak, 2 for moderate, 3 for high intensity). In cores where staining was of variable intensity the average intensity was reported. Each array was independently analysed in a blind study by two independent observers. Inter-rating correlation was >95%. When strong differences in scoring between the two observers (more than 1 unit per core) occurred the core was re-evaluated to reach a concordant scoring between the two observers. The average of cancerous scores of the same patient was used for analysis. No decrease in staining intensity on older paraffin blocks was observed.

### Statistical analysis

Statistical analysis was performed with SPSS software 11.0 (SPSS Inc. Chicago, IL, USA). To avoid a bias due to different efficiency of antibody hybridisation on different array slides we calibrated each slide for interslide comparison. The Spearman correlation coefficient test (two tailed) was used to estimate the correlation with clinicopathologic variables. Prostate-specific antigen recurrence-free survival curves were plotted using the Kaplan–Meier analysis and the log-rank test was used to test for significant differences. Receiver operative characteristic (ROC) curves were used to determine the threshold value for each Akt marker. Cox univariate and multivariate proportional hazard models were used to estimate the hazard ratios for each marker. Multivariate analysis was performed using a forward stepwise hazard model on univariate analysis required for entry into the model. Categorical variables for the model included surgical margins, extracapsular invasion status, perineural infiltration, preoperative PSA level, Gleason score, stage, grade and lymph node metastases.

### Western blot analysis

Cells were lyzed with cold lysis buffer (10 mM Tris-HCl, pH 7.4, 150 mM NaCl, 1 mM EDTA, 1 mM DTT/1 mM NaF/0.5% NP-40/0.5 mM PMSF/0.2 mM sodium orthovanadate/2 *μ*g ml^−1^ of aprotinin, leupeptin and pepstatin), boiled in loading buffer, separated by 10% SDS–PAGE, and transferred on a nitrocellulose membrane under refrigerated conditions (200 mA, 2 h). Membranes were saturated with 5% milk/TBS/0.1% Tween-20. Immunodetection was performed as described in the protocol of the ECL kit (Amersham Pharmacia, Amersham Biosciences, Backinghamshire, UK): that is incubated 2 h at room temperature with the specific Akt antibodies (1 to 2 *μ*g ml^−1^), washed two times with TBS/0.1% Tween-20 and incubated for another 30 min at room temperature with peroxidase-conjugated antibodies (Santa-Cruz Biotechnology, CA, USA).

## RESULTS

### Akt expression and localisation in normal and cancer tissues

**Figure 1:**
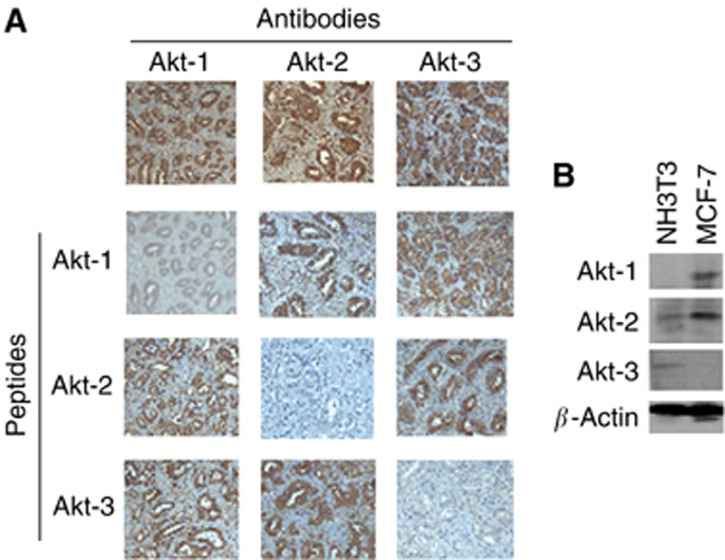
Validation of Akt-specific antibodies by IHC and Western blot. (**A**) Immunohistochemistry staining was performed using antibodies against Akt-1, Akt-2, Akt-3 with or without corresponding and not corresponding blocking peptides as indicated. Notice that the specific peptide for the corresponding antibody abolishes or reduces the staining of cancerous areas while the nonspecific peptides reduce the staining only in stroma areas but not in cancerous areas. (**B**) Expression of Akt in different cell lines: NIH 3T3 show low expression of Akt-1 and Akt-2 but higher expression of Akt-3 while MCF-7 and LNCaP cells show no expression of Akt-3 but expression of Akt-1 and Akt-2. Cell lysates were subjected to Western blotting and revealed using specific Akt isoform antibodies as indicated.

**Figure 2:**
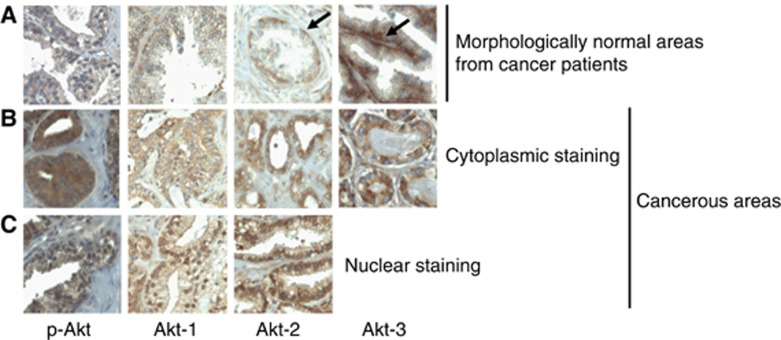
Expression and localisation of Akt-1, -2 and -3 in prostate cancer tissues. Immunohistochemistry staining was performed using specific antibodies against Akt-1, Akt-2, Akt-3 or phospho-Akt as indicated. (**A**) Staining normal. (**B**) Cytoplasmic expression in cancerous areas. (**C**) Nuclear staining in cancerous areas. Notice the basal cell staining by anti-Akt-1 and anti-Akt-2 antibodies. Magnification × 40. Arrows show basal cell staining.

In total, 373 prostate cancer tissues from 63 cancer prostate patients spotted on tissue microarrays (Table 1) were evaluated for Akt-1, -2, -3 expressions by IHC using isoform-specific antibodies. In agreement with previous studies (Vasko *et al*, 2004; Kirkegaard *et al*, 2005) the specificity of each antibody has been confirmed by IHC using blocking peptides (Figure 1A) and by Western blot by comparing NIH 3T3 cells, known to express low level of Akt-1 and Akt-2 but strong level of Akt-3, with MCF-7 cells that do not expressed Akt-3 (Figure 1B). Non-neoplasic (*n*=141) and cancerous (*n*=232) cores were separately examined. The three isoforms were expressed in all normal and cancerous tissues with the exception of one patient showing no expression of Akt-1 and Akt-2 but weak expression of Akt-3, and another patient showing no expression of Akt-3 and weak expression of Akt-2 and Akt-1. Akt isoforms were mainly localised in the cytoplasm and membrane. In addition, several tissues also presented nuclear localisation of Akt-1 and Akt-2 but not Akt-3 (Figure 2C). Nuclear localisation was independent of the cytoplasmic intensity of staining. In addition to this general pattern, Akt-3 and less often Akt-2, were also strongly expressed in the basal cell layer (Figure 2A).

Independent of localisation, the three isoforms were significantly overexpressed in cancerous areas as compared to adjacent non-neoplasic areas (*P*<0.001, Students’ *t*-test). Approximately 60, 65 and 59% of cancerous tissues expressed high levels of Akt-1, -2, -3, respectively, compared to normal adjacent cores while 30% of normal tissue cores showed a high expression of either Akt-1, Akt-2 or Akt-3. In addition, in normal and cancerous cores a strong correlation between expression of phospho-Akt and the three isoforms was also observed (*r*=0.70, 0.75, 0.65, *P*<0.001, Spearman's test).

### Correlation between cytoplasmic Akt in cancerous tissues and clinical parameters

We assessed the correlation between the high expression of each Akt isoform and clinicopathologic parameters available for patients: Gleason score, surgical grade, pathological stage, extracapsular invasion, perineural infiltration, preoperative PSA level, PSA relapse time, hormone-refractory cancer progression after surgery and nodal metastasis status at time of surgery (Table 1). Overexpression of Akt was determined to be cases that stained above the median expression of cancerous cores on the tissue array. First, we analysed the cytoplasmic staining. As shown in Table 2, in cancerous tissues, no correlation was observed between Akt-1, -2, or -3 and Gleason score, surgical grade or pathological stage. Akt-1 expression tended to positively correlate with preoperative PSA level (*r*=0.24, *P*=0.06, Spearman's test). However, a significant stronger Akt-1 staining was obtained in patients with preoperative PSA>10 ng (*P*=0.046, Mann–Whitney *U*-test). We also noticed a weak trend of cytoplasmic Akt-1 expression to correlate inversely with PSA recurrence-free interval (*r*=−0.28, *P*=0.10, Spearman's test).

Although, no correlation was observed between cytoplasmic Akt-2 and clinical parameters tested (Table 2), cytoplasmic Akt-3 correlated with resistance to hormone therapy (*r*=0.34, *P*=0.01, Spearman's test) and showed a tendency to correlate with extracapsular invasion (*r*=0.23, *P*=0.07, Spearman's test). A correlation between cytoplasmic Akt-3 and PSA relapse interval was also observed (*r*=−0.23) but was not significant (*P*=0.20).

### Correlation between nuclear Akt in cancerous tissues and clinical parameters

Inversely to its cytoplasmic expression, the nuclear staining of Akt-1 in cancerous tissues directly correlated with PSA relapse time (*r*=0.40, *P*=0.02, Spearman's test, Table 2), and negatively with perineural infiltration (*r*=−0.38, *P*<0.01). In contrast, nuclear Akt-2 in cancerous tissues was not significantly associated with any parameter tested, but only showed a trend to correlate inversely with perineural infiltration and lymph node invasion (*r*=−0.22, *P*=0.08, Table 2).

### Akt-1 and Akt-2 in cancerous tissues are associated with PSA relapse

**Figure 3:**
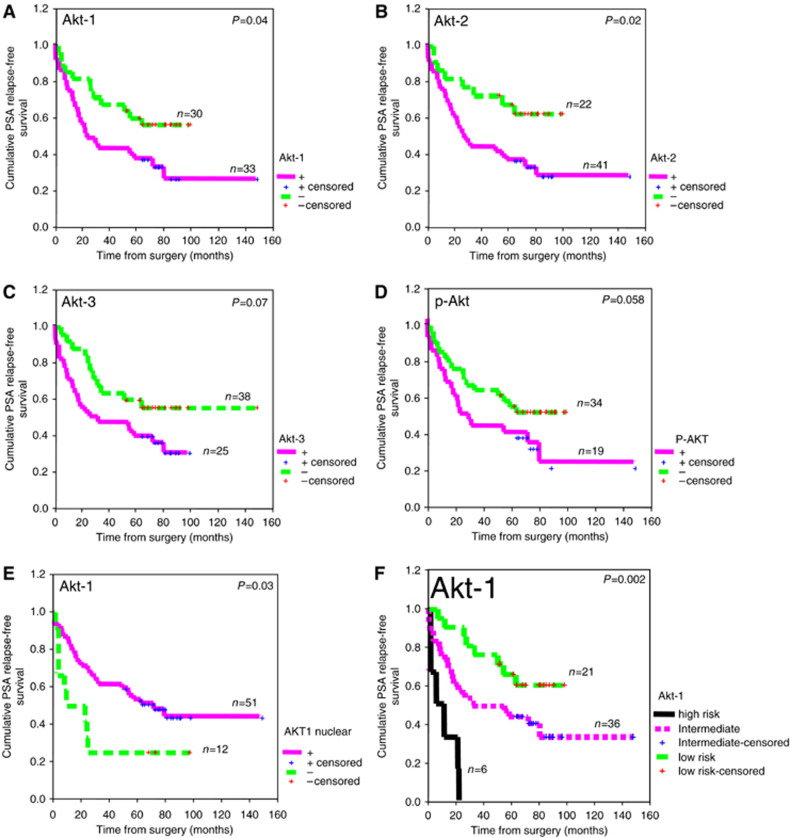
Kaplan–Meier PSA recurrence-free survival curve in patients with prostate cancer. *n*=Number of patients. Significance (*P*) is indicated by log rank (**A**) cytoplasmic Akt-1 in cancerous tissues. (**B**) cytoplasmic Akt-2 in cancerous tissues in cancerous tissues. (**C**) Cytoplasmic Akt-3 in cancerous tissues. (**D**) Cytoplasmic phospho-Akt. (**E**) Nuclear Akt-1 in cancerous tissues. (**F**) High-risk patients (high cytoplasmic Akt-1/absence of nuclear Akt-1), intermediate risk patients (high cytoplasmic Akt-1/nuclear Akt-1 and low cytoplasmic Akt-1/absence of nuclear Akt-1), low-risk patients (low cytoplasmic Akt-1/Akt-1 nuclear).

As overexpression of cytoplasmic Akt-1 and Akt-3 in cancerous tissues was correlated with PSA recurrence-free interval, we performed Kaplan–Meier analysis and Cox proportional hazard model to estimate the association between overexpression of Akt-1, Akt-2 or Akt-3 and PSA relapse. For comparison purpose, we also considered expression of the active form of Akt (phospho-Akt) that has previously been described as a significant predictor of PSA relapse (Ayala *et al*, 2004; Kreisberg *et al*, 2004). Receiver operative characteristic curves were used to estimate an optimal threshold values for the cytoplasmic markers that could be used to predict PSA failure. Nuclear Akt-1 and Akt-2 were categorised by their presence or absence. Consistent with the correlations previously observed in cancerous tissues, the overexpression of cytoplasmic Akt-1 was significantly associated with earlier PSA relapse (*P*=0.04, log rank test, Figure 3A and Table 3). In addition, Akt-2 was also significantly associated with earlier PSA relapse (*P*=0.02, log rank test, Figure 3B and Table 3) while Akt-3 and phospho-Akt were borderline significant (*P*=0.07, *P*=0.06, respectively, log rank, Table 3, Figure 3C and D). In univariate analysis a slightly increased risk of relapse was associated with these markers (Table 4). Akt-2 showed a slightly higher risk of relapse than Akt-1 (Table 4).

Nuclear Akt-1 in cancerous tissues was significantly associated with a longer interval for PSA relapse (*P*=0.03, log rank test, Table 3), but not nuclear Akt-2. The median relapse time of patients without nuclear Akt-1 was 32 months compared to 82 months for patients with nuclear Akt-1 (Figure 3E). The 2.24-fold decrease in the risk of relapse (hazard ratio, *P*=0.05, Table 4) confirmed a protective effect of nuclear Akt-1.

As no significant correlation was observed between the presence of nuclear Akt-1 and the intensity of Akt-1 cytoplasmic staining (*P*=0.67, *r*=0.05, Spearman's test), we tested the hypothesis that combining both markers may be more informative and help predict risk group. A high-risk patient group (high cytoplasmic Akt-1 and absence of protective nuclear Akt-1), a low-risk patient group (low Akt-1 cytoplasmic and the presence of protective nuclear Akt-1) and an intermediate group composed of the remaining patients. As predicted, the high-risk patient group showed a significant lower mean PSA relapse interval (13 months), while the low-risk patients showed a significant higher mean PSA relapse interval (73 months) compared to the intermediate group (68 months) (*P*=0.002, log rank test, Figure 3F). In another words all high-risk patients had a PSA recurrence before 2 years while intermediate risk patients had recurrence with variable interval (40% before 2 years) and most low-risk patients (20 out of 22) had PSA relapse after 2 years.

In multivariate analysis, when representative prognostic variables of univariate significance were undertaken (surgical margins, extracapsular invasion, perineural infiltration, Gleason score, lymph node invasion and pathologic stage and surgical grade, PSA level and Akt markers), only the combination of cytoplasmic and nuclear Akt-1 markers, Gleason score and stage remained independent predictive variables of PSA relapse with the pathologic stage showing the highest risk of relapse (Table 4).

### Akt is not associated with survival

In addition to the association with PSA-relapse, we also tested the association between each isoform of Akt and survival. Kaplan–Meier analysis and Cox regression hazard model demonstrated no significant association between Akt and survival given the relatively low mortality rate in this cohort of radical prostatectomy patients (Figure 3 and Table 4).

## DISCUSSION

In men, prostate cancer is the most frequently diagnosed cancer and a leading cause of cancer death. Unfortunately, the cellular events responsible for progression to hormone-independent prostate cancer are not yet well known. There are no reliable molecular markers that predict if a cancer will progress to an aggressive disease. Recently, attention has focused on the activity of the protein kinase Akt due to its role in cell survival, proliferation, apoptosis and its association with PSA relapse in prostate cancer patients (Graff, 2002). However, the precise involvement of Akt in prostate cancer progression is not well defined and in particular it is not known if all three isoforms are associated with disease progression and have the same role in prostate cancer progression. Here, we investigated the correlation between the level of each Akt isoform and different clinical parameters. Even if the signalling role of Akt is exerted only when the kinase is activated by phosphorylation, we expect that the amount of each isoform reflects their respective activity level since no differential affinity of the upstream activator PDK for the different Akt has been reported to date. The correlation between levels of expression and downstream activation is further supported by *in vitro* studies in cell lines (Nakatani *et al*, 1999).

Interestingly, we found that the expression of Akt-1, Akt-2 and Akt-3, did not correlate with the same clinical parameters suggesting that different Akt isoforms may have different and specific roles. This hypothesis is reinforced by the fact that the phospho-Akt staining, which is unable to dissociate the different isoforms, did not show any strong correlation with these parameters. Similar results have recently been reported where only a weak correlation between phospho-Akt expression and clinical stage was observed (Ayala *et al*, 2004). In addition, our study found that Akt-1 and phospho-Akt overexpression in cancerous tissues was correlated with a high preoperative PSA level. This result is consistent with a previous study where a significant difference in the intensity of Pan-Akt staining in tumours from patients with high and low PSA level was observed (Liao *et al*, 2003). In contrast, Akt-3 expression correlated with extracapsular invasion and hormone-refractory disease progression, suggesting that Akt-3 is involved in the invasion potential of prostate cancer cells, and may therefore play a role in later stages of the disease. More *in vitro* and *in vivo* studies are needed to support this hypothesis. In our cohort, the number of patients progressing to a hormone-refractory disease was relatively small and the role of Akt-3 in hormone therapy resistance should be estimated on a larger scale.

Surprisingly, we also found an inverse correlation between nuclear and cytoplasmic Akt-1 and clinical parameters. High cytoplasm expression of Akt-1, and also Akt-3, was correlated with poor prognosis parameters, such as elevated preoperative PSA levels, earlier PSA relapse, hormone-refractory disease progression, extracapsular invasion while the presence of Akt-1, and Akt-2, in the nucleus of normal or cancerous tissues was correlated with better prognosis parameters such as later PSA relapse and absence of perineural infiltration. Similarly, the presence of nuclear Akt has also been correlated with good prognosis in lung cancer, endometrial carcinoma and superficial spreading melanoma (Shah *et al*, 2005; Slipicevic *et al*, 2005; Uegaki *et al*, 2005). These observations are reinforced by the combined effect of nuclear and cytoplasmic Akt-1 markers on recurrence disease, as seen on Figure 3. These results suggest that compartementalisation of Akt may be important in determining its cellular effect. In different cancers, activation of Akt acts by phosphorylation of transcription factors, signalling components such as IKK, caspase 9, mTor, Bad and others. Although most of these targets are phosphorylated in the cytoplasm, some appear to be phosphorylated in the nucleus (Liang *et al*, 2002; Nicholson and Anderson, 2002; Shin *et al*, 2002; Viglietto *et al*, 2002). In prostate cancer, Akt has been shown to modulate by phosphorylation the activity and stabilisation of the nuclear androgen receptor (Lin *et al*, 2001, 2002). It is thus tempting to speculate that nuclear Akt-1 or Akt-2 can phosphorylate nuclear AR to reduce its expression and androgen growth response of cells. In contrast, when Akt is absent from the nucleus higher AR level can induce androgen-dependent growth favouring disease progression, which would correlate with a shorter disease relapse as observed in the present study.

The prognostic significance of each isoform of Akt has not been examined in other cancers from chemotherapy-free patients. In contrast phospho-Akt has been examined in a number of other cancers, although different results were obtained depending of the cancer tissues examined. For example, in breast, renal and head and neck carcinoma, phospho-Akt is associated with cancer recurrence (Perez-Tenorio and Stal, 2002; Horiguchi *et al*, 2003; Kirkegaard *et al*, 2005). In gliomas, and ovarian cancer no association between phospho-Akt and survival has been observed (Ermoian *et al*, 2002; Wang *et al*, 2005). In prostate cancer phospho-Akt has also been associated with poor outcome (Ayala *et al*, 2004; Kreisberg *et al*, 2004). In contrast to our observations, these two studies described phospho-Akt as a strong predictor of disease recurrence. While we also observed an association between phospho-Akt expression and PSA relapse in our study this association was weak. Difference in choice of patient cohorts easily explains these results since here we included a considerable number of patients with positive surgical margin (50% of our cohort) and the follow-up of our patients was longer (over 5 years). Altogether, our study is in agreement that phospho-Akt is a predictor of recurrence but seems to be a weaker predictor than Akt-1 and than pathological parameters such as surgical margins or pathological stage.

Our study shows a differential role for each isoform of AKT in the progression of prostate cancer. Based on this data we propose that Akt may play an important role in the biology of prostate cancer progression and suggests that Akt may eventually become a promising therapeutic target.

## Figures and Tables

**Table 1:** Prostate cancer patients cohort

Age median (min–max)	62 (49–70)
	
*Stage*	
Stage 2	34
Stage 3	29
	
*Invasion*	
Extracapsular invasion	19
Lymph node invasion	9
Perineural infiltration	9
	
Hormone refractory disease	5
Prostatis	1
	
*Gleason score*	
Gleason 4	7
Gleason 5	14
Gleason 6	14
Gleason 7	18
Gleason 8–9	10
	
*Preoperative PSA*	
<10 ng	35
>10 ng	25
Not available	2
	
*PSA relapse*	
Relapse	35
No relapse	28
	
*Surgical margins*	
Negative	31
Positive	32
Survival	54

**Table 2:** Spearman correlation test (two tailed) between Akt expression in cancerous tissues and clinical parameters

	**Cytoplasmic**								**Nuclear**					
	**Akt1**		**Akt2**		**Akt3**		**p-Akt**		**Akt1**		**Akt2**		**p-Akt**	
**Spearmann**	***r***	***P***	***r***	***P***	***r***	***P***	***r***	***P***	***r***	***P***	***r***	***P***	***r***	***P***
Hormone-refractory disease	0.02	0.87	−0.06	0.62	**0.34**	**0.01**	−0.05	0.73	−0.01	0.96	−0.05	0.71	−0.18	0.15
Preoperative PSA	**0.24**	**0.06**	0.09	0.48	0.11	0.4	**0.28**	**0.03**	0.09	0.5	0.22	0.09	−0.03	0.82
Gleason score	−0.046	0.72	−0.16	0.21	0	1	−0.07	0.58	−0.15	0.23	−0.1	0.56	−0.05	0.7
Extracapsular invasion	−0.067	0.6	0.09	0.47	**0.23**	**0.07**	−0.07	0.57	−0.03	0.79	−0.09	0.43	0.16	0.2
PSA relapse	**−0.28**	**0.1**	0.07	0.68	−0.23	0.2	0.17	0.33	**0.4**	**0.02**	−0.03	0.86	−0.09	0.5
Stage	−0.04	0.76	−0.05	0.72	0.17	0.18	−0.12	0.37	−0.12	0.35	−0.08	0.54	0.08	0.53
Grade	−0.07	0.59	−0.12	0.34	−0.07	0.59	−0.07	0.59	−0.15	0.25	−0.14	0.27	−0.07	0.59
Survival	0.07	0.6	0.04	0.76	−0.1	0.42	−0.12	0.36	−0.02	0.9	−0.07	0.58	0.02	0.87
Lymph node invasion	−0.03	0.83	0.04	0.76	−0.1	0.42	−0.21	0.1	−0.033	0.8	**−0.22**	**0.08**	0.04	0.76
Perineural infiltration	0.16	0.21	−0.14	0.27	−0.1	0.42	0.16	0.21	**−0.38**	**<0.01**	**−0.22**	**0.08**	0.04	0.76

*r*=correlation coefficient. Correlation *r*>0.20 are indicated in bold.

**Table 3:** Significance of Akt staining and association with PSA relapse or survival

			**PSA relapse**		**Survival**	
**Tissue**	**Marker**	**Localization**	***P***	*P* ^*^	***P***	*P* ^*^
Cancer	Akt1	Nucleus	0.03**	**0.05**	0.68	0.69
		Cytoplasm	0.04**	**0.04**	0.72	0.73
						
	Akt2	Nucleus	0.74	0.74	0.43	0.46
		Cytoplasm	0.02**	**0.03**	0.79	0.79
						
	Akt3	Nucleus	NA	NA	NA	NA
		Cytoplasm	0.07**	0.08	0.85	0.85
						
	p-Akt	Nucleus	0.88	0.88	0.35	0.35
		Cytoplasme	0.06**	0.06	0.64	0.64
						
	Combined Akt1	Nucleus/cytoplasmic	**0.002**	**0.001**	0.49	0.99

*P*=log rank test.

P^*^=univariate Cox regression model; PSA=prostate-specific antigen.

** Kaplan–Meier graph are shown in Figure 3. Bold values highlight significant association.

**Table 4:** Prognostic factors predicting PSA relapse in prostate cancer cohort of 63 patients

**PSA relapse**	**Hazard ratio (95% CI)**	***P***
*Univariate*		
Stage	5.419 (2.627–11.179)	<0.001
Grade	2.025 (1.193–3.436)	0.009
LN invasion	2.02 (0.88–4.634)	0.097
Preneural infiltration	2.250 (0.982–5.153)	0.055
Extracapsular invasion	3.332 (1.713–6.481)	<0.001
Gleason score	1.514 (1.168–1.963)	0.002
Margins	5.372 (2.498–11.551)	<0.001
Akt1 cytoplasmic	2.032 (1.014–4.074)	0.05
Akt1 nuclear	2.242 (1.047–4.808)	0.04
Akt2 cytoplasmic	2.406 (1.094–5.290)	0.03
Akt3 cytoplasmic	1.908 (0.938–3.883)	0.08
p-Akt cytoplasmic	1.867 (0.964–3.612)	0.06
Akt-1 cytoplasmic/nuclear	2.722 (1.496–4.953)	0.001
		
*Multivariate*		
Stage	3.581 (1.63–7.87)	<0.001
Akt-1 cytoplasmic/nuclear	2.118 (1.12–3.99)	0.02
Gleason score	1.357 (1.01–1.82)	0.04

Cox regression models. CI=confidence interval; PSA=prostate-specific antigen. Akt staining corresponds to staining in cancerous tissues.
